# Video-Assisted Thoracoscopic Repair of an Iatrogenic Innominate Arterial Injury

**DOI:** 10.1093/icvts/ivag112

**Published:** 2026-04-11

**Authors:** Xinyu Wang, Zhiyong Sun, Yujie Fu, Xiaojing Zhao

**Affiliations:** Department of Thoracic Surgery, Renji Hospital, Shanghai Jiao Tong University School of Medicine, Shanghai 200127, China; Department of Thoracic Surgery, Renji Hospital, Shanghai Jiao Tong University School of Medicine, Shanghai 200127, China; Department of Thoracic Surgery, Renji Hospital, Shanghai Jiao Tong University School of Medicine, Shanghai 200127, China; Department of Thoracic Surgery, Renji Hospital, Shanghai Jiao Tong University School of Medicine, Shanghai 200127, China

**Keywords:** video-assisted thoracic surgery, innominate arterial injury, vascular repair

## Abstract

We report a 64-year-old male with iatrogenic innominate artery haemorrhage during video-assisted thoracic surgery (VATS) lymph node dissection, who underwent successful thoracoscopic repair. For centres with experienced minimally invasive thoracic surgeons and rapid multidisciplinary support, thoracoscopic repair of such an injury is feasible and can avoid thoracotomy.

## INTRODUCTION

Intraoperative injuries to systemic arteries (eg, innominate or subclavian artery) during VATS are rare but fatal due to rapid haemorrhage and limited operative space. Urgent thoracotomy is most commonly used for massive bleeding in VATS lung surgery, while thoracoscopic repair is a specialized alternative reserved for centres and surgeons with advanced expertise.^1^ We present a case of thoracoscopic repair for iatrogenic innominate arterial injury and summarize its technical essentials.

## CASE PRESENTATION

**Figure 1.**
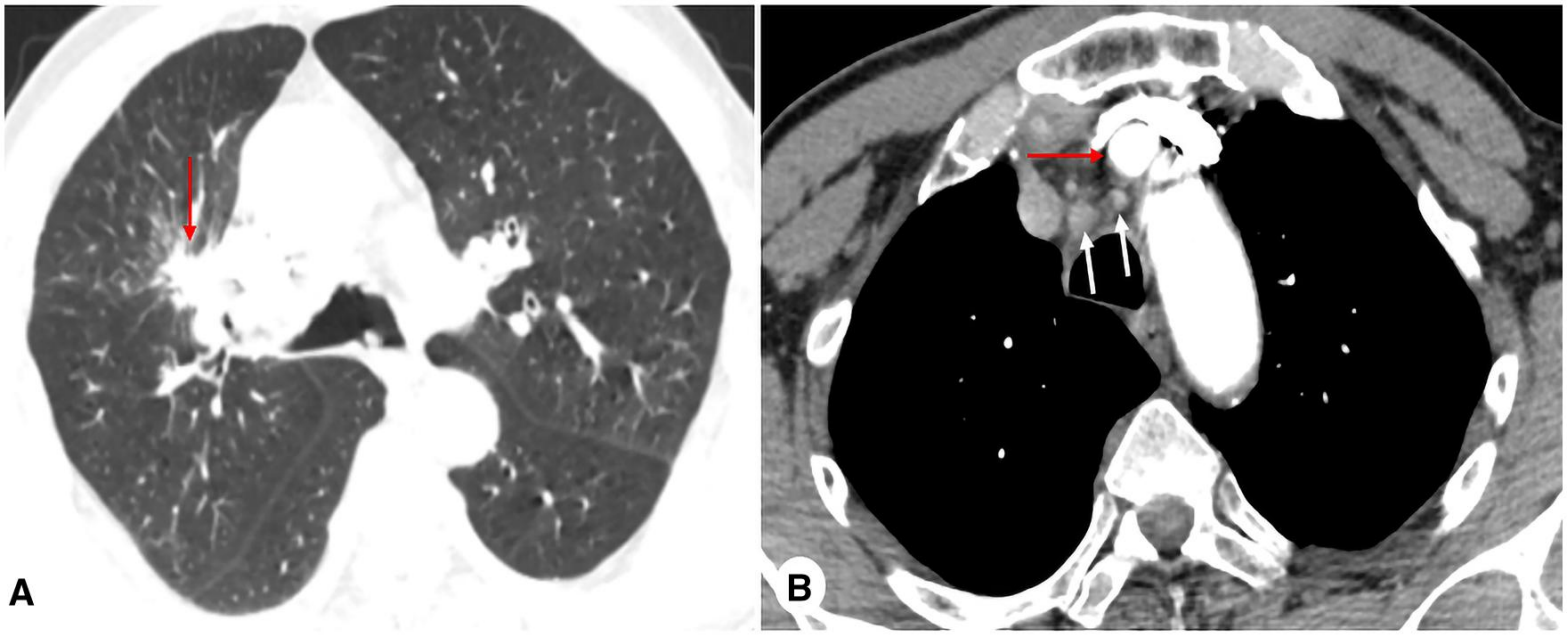
(A) CT scan showing the primary right upper lobe lesion (single arrow). (B) Mediastinal window showing the innominate artery (single arrow) and its close proximity to mediastinal lymph node station 2 R (two arrows).

**Figure 2.**
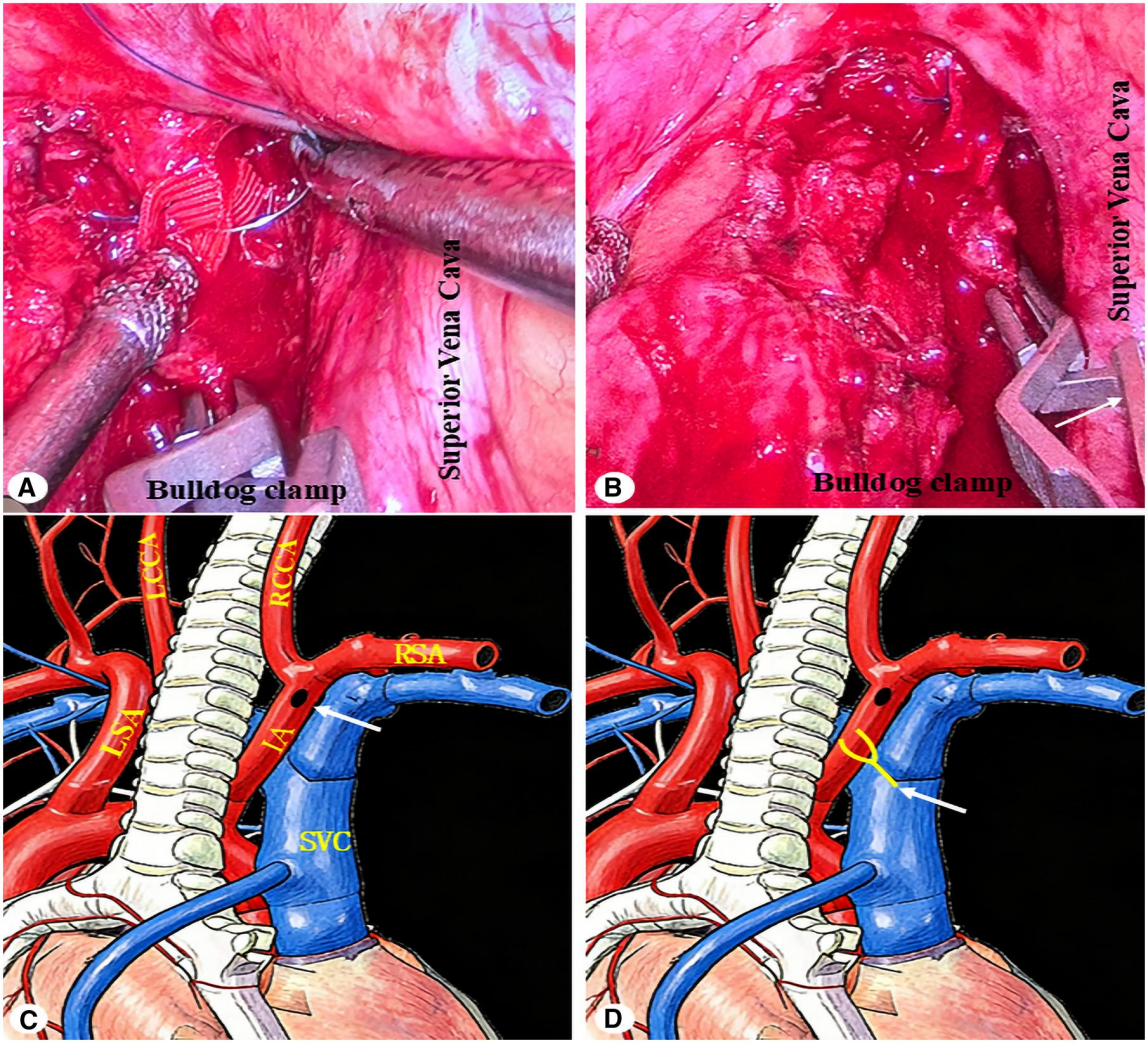
(A) Intraoperative image of innominate artery suturing. (B) Intraoperative image of achieved haemostasis. (C) Schematic of the bleeding site (arrow). (D) Schematic of the clamping site (arrow).

A 64-year-old man was diagnosed with cT3N2M0 central squamous cell carcinoma of the right upper lung and received 4 cycles of neoadjuvant chemo-immunotherapy. Mediastinoscopic sampling of station 4R/4L lymph nodes showed no metastasis, and he was referred for surgical resection following restaging chest CT (Figure 1). On September 4, 2025, uniportal VATS right upper lobectomy was performed initially, followed by sequential systemic lymphadenectomy (stations 10, 11, 12, 7, 4R, 2R). Sudden brisk bleeding occurred during station 2R dissection due to inadvertent harmonic scalpel trauma, suggesting an innominate artery lateral-wall laceration. Sterile gauze was used for temporary haemostasis, 2 additional 1.5-cm ports at the right anterior and posterior axillary lines at the 7th intercostal space were created for suturing, and urgent multidisciplinary consultation (anaesthesiology, cardiac surgery) was initiated. The proximal innominate artery was occluded with a bulldog clamp, and a ∼1.0 cm lateral defect was identified after haematoma evacuation. It was primarily repaired with 3 interrupted 4–0 Prolene U-stitches reinforced with felt pledgets, achieving complete haemostasis (Figure 2). The total operative duration was 270 min, comprising 40 min for injury repair and 25 min of vascular clamping. Estimated blood loss was 1500 mL, with intraoperative transfusion of 4 U packed red blood cells and 600 mL fresh frozen plasma.

The patient was extubated 8 hours postoperatively in the ICU. Following a 2-day ICU stay, he was transferred to the general ward, with hourly neurological and right upper extremity perfusion monitoring. He had a mild, transient Miller Grade I air leak without recurrent bleeding, neurological deficits, or vascular complications and was discharged on postoperative day 12. Final pathology confirmed complete tumour regression (ypT0N0M0, TRG 0) with R0 resection, and all 15 harvested lymph nodes were metastasis-free. At 3-month follow-up, he recovered well without sequelae, and duplex ultrasound of the innominate artery confirmed no evidence of pseudoaneurysm formation or stenosis.

## DISCUSSION

Injury contributors included mediastinoscopic sampling and neoadjuvant therapy-induced fibrosis/adhesions obscuring tissue planes. Thoracoscopic repair of innominate artery injury is rarely documented compared with left subclavian artery injury.^2^^,^^3^ This case confirms its feasibility with advanced thoracoscopic suturing skills, avoiding thoracotomy. Key management principles are summarized as follows:

### Rapid temporary haemostasis and multidisciplinary activation

Immediate gauze tamponade prevents exsanguination. Anaesthesia should secure large-bore venous access, prepare blood products, and implement cerebral protection. Early consultation with vascular/cardiac surgery and interventional radiology is recommended. Stent-grafting is technically feasible for innominate artery injury but requires on-site endovascular capability and careful assessment of branch vessel coverage.^4^ The injury in our case was a localized laceration evaluated by the multidisciplinary team, which agreed on the thoracoscopic repair strategy rather than stent-grafting.

### Proximal vascular control and repair technique

Proximal control of high-pressure systemic arteries should be obtained prior to definitive repair, which can be achieved using atraumatic vascular clamps (eg, bulldog clamps). Intermittent clamp release preserves distal perfusion and alleviates ipsilateral cerebral and limb ischaemia. Felt-buttressed interrupted sutures ensure secure closure and reduce suture-tearing risk. Additionally, 4–0 Prolene fine monofilament non-absorbable sutures are preferred, and overtightening should be avoided to prevent arterial luminal stenosis. Real-time monitoring of cerebral tissue oxygenation by near-infrared spectroscopy (NIRS) is recommended during vascular repair.^5^

### Decision to convert to thoracotomy

Thoracoscopic repair of iatrogenic innominate arterial injuries is only feasible for simple and localized lacerations. Prompt thoracotomy is needed for an inadequate operative field, uncontrollable bleeding, vessel transection requiring grafting, or unsafe anatomical exposure, as open surgery enables faster and more secure vascular reconstruction in such circumstances.

## Data Availability

The data in this study are available from the corresponding author.
